# Applicability of the surgical risk calculator by the American College of Surgeons in the setting of German patients undergoing complete pancreatectomy: multicentre study using data from the StuDoQ|Pancreas registry

**DOI:** 10.1093/bjsopen/zrac164

**Published:** 2023-03-07

**Authors:** Philipp Höhn, Fabian Runde, Andreas Minh Luu, Tim Fahlbusch, Daniel Fein, Carsten Klinger, Waldemar Uhl, Orlin Belyaev, members of StuDoQ|Pancreas registry of the German Society for General and Visceral Surgery (DGAV), Carsten Gutt, Jörg Köninger, Andreas Schnitzbauer, Clemens Schafmayer, Stefan Farkas, Werner Hartwig, Sören Torge Mees, Frank Klammer, Matthias Glanemann, Michael Ghadimi, Matthias Anthuber, Christoph Reißfelder, Pompiliu Piso, Winfried Padberg, Robert Grützmann, Marco Niedergethmann, Andreas Pascher, Klaus Prenzel, Hans-Bernd Reith, Ansgar Michael Chromik, Colin M Krüger, Hüseyin Bektas, Bertram Illert, Merten Hommann, Jörg-Peter Ritz, Axel Döhrmann, Nico Schäfer, Thomas Kraus, Mark Jäger, Jörg Tschmelitsch, Ullrich Fleck, Michael Pauthner, Ute Tröbs, Albrecht Stier, Carsten Krones, Tobias Keck, Jens Werner, Natascha Nüssler, Detlef K Bartsch, Christoph-Thomas Germer, Helmut Friess, Christian Mönch, Karl-Jürgen Oldhafer, Jörg C Kalff

**Affiliations:** Department of General and Visceral Surgery, St. Josef-Hospital, Ruhr-Universität Bochum, Bochum, Germany; Faculty of Medicine, Ruhr-Universität Bochum, Bochum, Germany; Department of General and Visceral Surgery, St. Josef-Hospital, Ruhr-Universität Bochum, Bochum, Germany; Department of General and Visceral Surgery, St. Josef-Hospital, Ruhr-Universität Bochum, Bochum, Germany; Department of General and Visceral Surgery, St. Josef-Hospital, Ruhr-Universität Bochum, Bochum, Germany; StuDoQ Registry, German Society for General and Visceral Surgery, Berlin, Germany; Department of General and Visceral Surgery, St. Josef-Hospital, Ruhr-Universität Bochum, Bochum, Germany; Department of General and Visceral Surgery, St. Josef-Hospital, Ruhr-Universität Bochum, Bochum, Germany; Surgical Department, Universitätsklinikum Schleswig-Holstein Campus Lübeck, Lübeck, Germany; Department of General, Visceral, Transplant, Vascular and Thoracic Surgery, Klinikum Großhadern, Ludwig-Maximilians-Universität München, München, Germany; Department of General and Visceral Surgery, Endocrine Surgery und Coloproctology, Klinikum Neuperlach, Städt. Klinikum München, München, Germany; Department of Visceral, Thoracic and Vascular Surgery, Universitätsklinikum Marburg, Marburg, Germany; Department of General, Visceral, Vascular and Pediatric Surgery, Universitätsklinik Würzburg, Würzburg, Germany; Department of Surgery, Klinikum rechts der Isar, Technische Universität München, München, Germany; Department of General, Visceral and Transplant Surgery, Westpfalz-Klinikum Kaiserslautern, Kaiserslautern, Germany; Department of Surgery, Asklepios Klinik Barmbek, Barmbek, Germany; Department of Visceral, Colorectal Surgery and Proctology, Universitätsklinikum Bonn, Bonn, Germany

## Abstract

**Introduction:**

Surgical risk calculators can estimate risk probabilities for postoperative outcomes utilizing patient-specific risk factors. They provide meaningful information for obtaining informed consent. The aim of the present paper was to evaluate the predictive value of the surgical risk calculators by the American College of Surgeons in German patients undergoing total pancreatectomy.

**Methods:**

Data for patients who underwent total pancreatectomy between 2014 and 2018 were acquired from the Study, Documentation, and Quality Center of the German Society for General and Visceral Surgery. Risk factors were entered manually into the surgical risk calculators and calculated risks were compared with actual outcomes.

**Results:**

Of the 408 patients analysed, predicted risk was higher in patients with complications except for the prediction of re-admission (*P* = 0.127), delayed gastric emptying (*P* = 0.243), and thrombosis (*P* = 0.256). In contrast, classification of patients into below, above, or average risk by the surgical risk calculators only produced meaningful results for discharge to nursing facility (*P* < 0.001), renal failure (*P* = 0.003), pneumonia (*P* = 0.001), serious complications, and overall morbidity (both *P* < 0.001). Assessment of discrimination and calibration showed poor results (scaled Brier scores 8.46 per cent or less).

**Conclusion:**

Overall surgical risk calculator performance was poor. This finding promotes the development of a specific surgical risk calculator applicable to the German healthcare system.

## Teaser

We evaluated the Surgical Risk Calculator of the American College of Surgeons in a national, multicentre collective of patients undergoing total pancreatectomy. Overall performance was poor, but results are probably biased by input data. Whether the Surgical Risk Calculator is applicable to an external cohort remains unclear.

## Introduction

Naming the risk of specific and general complications is mandatory in obtaining informed consent from patients undergoing surgery^1^. Moreover, national health systems increasingly tie financial compensations for surgical procedures to quality of care. Performance comparisons of care providers in this context have to account for preoperative risk factors in the respective patient collective^2^. In recent years, multiple surgical risk calculators (SRCs) have been developed^3–5^. They predict the risk of postoperative outcomes based on planned procedure and preoperative risk factors. The SRC developed by the American College of Surgeons (ACS) utilizes standardized clinical data from 1.4 million cases in 393 North American hospitals participating in the National Quality Surgical Improvement Programme (NSQIP) collected between 2009 and 2012^3,6^. It is available as a free-to-access online tool (https://riskcalculator.facs.org/RiskCalculator/).

**Fig. 1:**
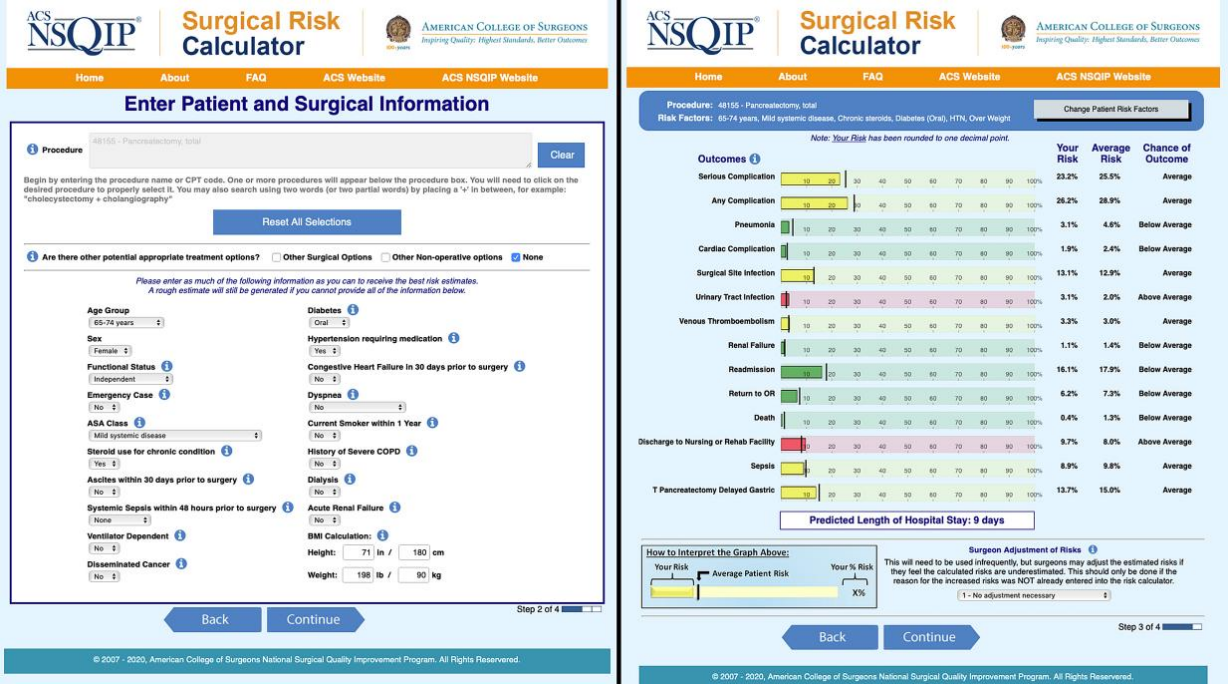
Front end of the surgical risk calculator (SRC) by the American College of Surgeons (ACS) Exemplary input of procedure and risk factors entered into the ACS SRC are shown on the left. The right side shows predicted risk for various outcomes and length of stay as calculated by the ACS SRC for the previously mentioned input. Reproduced with permission from the American College of Surgeons.

The ACS SRC accepts a planned procedure’s current procedural terminology (CPT) code and 19 patient-specific preoperative risk factors as inputs. The probability of nine different outcomes, two general groups (any complication and serious complication), and length of stay (LOS) is then predicted using a random intercept fixed-slope regression model. Risks are presented as percentages and chance of outcome (below average, average, and above average). Predicted risks (PRs) can be increased by one or two s.d. using a ‘surgeon adjustment of risks’ (SAR) menu. *Fig. 1* shows the input and output front end of the ACS SRC. Previous external validation attempts for NQSIP subgroups, international patient collectives, or specific procedures found mixed performance results^7–13^.

The German Study, Documentation and Quality Center (StuDoQ; Studien-, Dokumentations- und Qualitätszentrum) was initiated by the German Society of General and Visceral Surgery (DGAV; Deutschen Gesellschaft für Allgemein- und Viszeralchirurgie). Perioperative data on pancreatic surgery have been collected since 2013 and are mandatory for DGAV-certified centres for pancreatic surgery. Each collaborating hospital receives yearly quality reports based on the StuDoQ database. StuDoQ data can be obtained for research purposes by participating hospitals (*Supplementary material*). In 2020, 99 German hospitals provided data for the StuDoQ|Pancreas registry.

This study evaluates the ACS SRC’s applicability to a German multicentre patient collective undergoing complete pancreatectomy between 2014 and 2018 using data from the StuDoQ|Pancreas registry.

## Methods

### Data collection and processing

As recommended by the ACS SRC, a heterogenic multicentric data set was used^14^. Total pancreatectomy (CPT code 48155) was the chosen procedure because its variability in surgical technique is lower than in pancreatic resections with anastomosis, and morbidity and mortality are relatively high, allowing for more precise evaluation of postoperative outcomes^15^.

A total of 408 patients from the StuDoQ|Pancreas registry undergoing total pancreatectomy between 2014 and 2018 were included. Available data from the StuDoQ|Pancreas registry were compared with the definitions of preoperative risk factors and outcomes provided by the ACS SRC. In case of matching definitions, data were extracted unaltered from the registry and entered into the ACS SRC. The risk factors of congestive heart failure, dyspnoea, current smoker, and acute renal failure were not provided with matching definitions by the registry but were synthesized from other available data (*Supplementary material*). As no data on preoperative sepsis or ventilator dependency were provided by the registry, these risk factors were generally assumed as not present. Data on postoperative outcomes were processed accordingly. Information on postoperative sepsis and urinary tract infection was unavailable from the registry and was therefore excluded from analysis. All other outcomes were available with diverging definitions between the ACS SRC and the StuDoQ|Pancreas registry. Detailed information about definitions of risk factors, outcomes, and data processing are shown in *Table S1* (preoperative risk factors) and *Table S2* (postoperative outcomes).

Preoperative risk factors for each patient were entered into the ACS SRC. PR and chance of outcome (COO) for complications were manually recorded for each patient.

### Statistical analysis

**Fig. 2:**
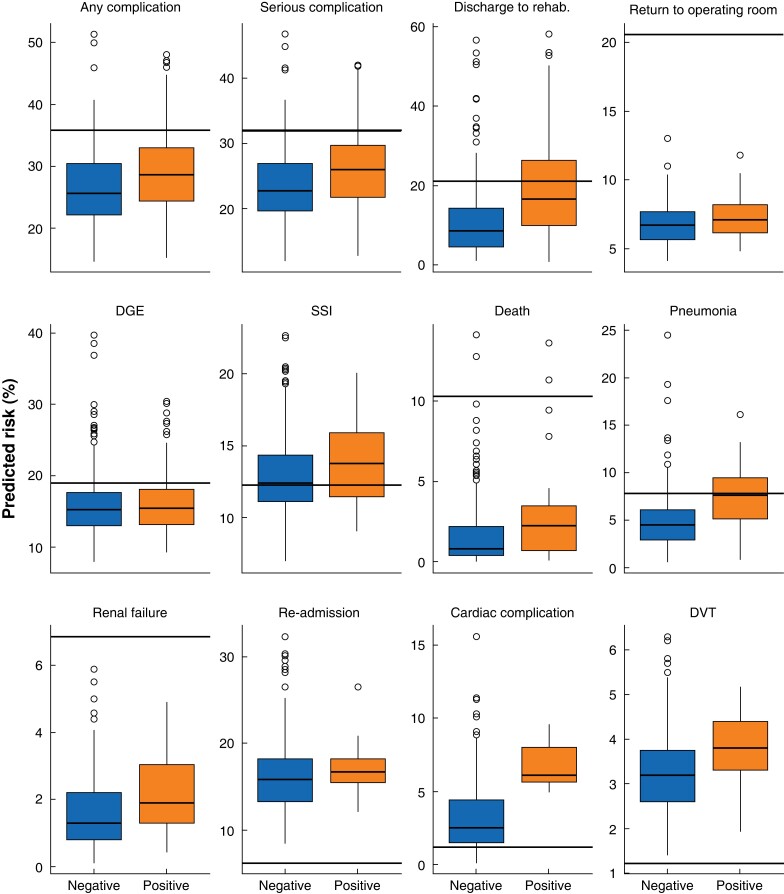
Predicted risk grouped by observed outcome Predicted risks of patients with observed outcome (positive) and without observed outcome (negative) are displayed as boxplots. Horizontal lines show observed outcome incidence. The y axis is scaled for each outcome. DGE, delayed gastric emptying; SSI, surgical site infection; DVT, deep venous thromboembolism.

Each predicted outcome was analysed separately. Patients were grouped according to occurrence of the analysed complication (positive) or non-occurrence (negative). PRs for each outcome in positive and negative patients are shown as boxplots in *Fig. 2*. Differences between central tendencies of PR between positive and negative patients were compared after normalization using a Mann–Whitney *U* test. Differences between predicted and actual LOS were compared in the same way. Occurrence of an outcome was compared with PR using a scaled Brier score (SBS) ranging between 0 and 100 per cent, with 100 per cent indicating a perfect prediction^16,17^.

Assigned COO was compared between positive and negative patients, creating a 2 × 3 contingency table that was analysed using Pearson chi-squared test. For sample sizes below five we used Fisher’s exact test. Results were considered statistically significant if *P* ≤ 0.050. Analysis was performed using Microsoft^®^ Excel (Microsoft Cooperation, Redmond, WA, USA), SPSS^®^ (IBM, Armonk, NY, USA), and RStudio (Posti Software, Boston, MA, USA). Further details are presented in *Supplementary material*.

### Ethics and data protection

Only anonymized data necessary for the described above analysis were provided by the StuDoQ|Pancreas Registry. Authors had no access to any identifying information and no data aggregation was performed. Written informed consent was obtained from all patients before data collection. Data extraction and analysis was cleared by the StuDoQ steering committee and performed in accordance with StuDoQ data protection guidelines (StuDoQ-2019-0010). Research was positively reviewed by the local ethics committee of Ruhr-University Bochum (20-6946-BR).

## Results

### Preoperative risk factors and patient characteristics

Data sets of 408 patients were included. The median age was 69 years. The male to female ratio was 1.18:1. The median BMI was 24.77 kg/cm². All procedures except one were elective (99.8 per cent). A total of 389 patients (95.4 per cent) were classified as ASA grade II or III. Hypertension requiring medication was the most common risk factor (61 per cent), followed by diabetes (33.8 per cent). A total of 77 patients (18.9 per cent) were classified as currently smoking and 29 patients (7.1 per cent) had a history of severe chronic obstructive pulmonary disease. All other risk factors were present in less than 3 per cent of patients (*Table 1*).

**Table 1:** Patient demographics and preoperative risk factors (*n* = 408)

Patient characteristics	
**Demographics**	
Sex ratio (M:F)	1.18:1
Age (years), median (minimum–maximum)	69 (34–87)
BMI (kg/m²), median (minimum–maximum)	24.77 (16.16–44.58)
Height (cm), median (minimum–maximum)	170 (148–198)
Bodyweight (kg), median (minimum–maximum)	72 (40–123)
**Functional status**	
Independent	389 (95.3)
Partially dependent	18 (4.4)
Totally dependent	1 (0.2)
**Emergency case**	
Elective	407 (99.8)
Emergency	1 (0.2)
**ASA score**	
ASA I	12 (2.9)
ASA II	175 (42.9)
ASA III	214 (52.5)
ASA IV	7 (1.7)
**Chronic steroid use**	5 (1.2)
**Ascites 30 days before surgery**	3 (0.7)
**Disseminated cancer**	11 (2.7)
**Diabetes**	
Insulin dependent	83 (20.3)
Oral medication	55 (13.5)
**Hypertension medication**	249 (61)
**Congestive heart failure**	9 (2.2)
**Dyspnoea with moderate exertion**	9 (2.2)
**Current smoker**	77 (18.9)
**Severe COPD**	29 (7.1)
**Dialysis**	3 (0.7)
**Postoperative duration of stay (days)**	
All patients, median (lower quartile–upper quartile; s.d.)	20 (9–31; 14.44)
Patients discharged at home, median (lower quartile–upper quartile; s.d.)	20 (10–30; 10.7)

Values are *n* (%) unless otherwise indicated. Cohort characteristics obtained from the StuDoQ|Pancreas registry as entered into the American College of Surgeons surgical risk calculator. Detailed definitions on all risk factors are provided in *Supplementary Table 1*. COPD, chronic obstructive pulmonary disease; StuDoQ, the German Study, Documentation and Quality Center.

### Observed postoperative outcomes

The most common observed outcome was unplanned return to the operating room (20.6 per cent), followed by delayed gastric emptying (18.9 per cent), and surgical site infection (SSI; 12.3 per cent). Eighty-six patients (21.1 per cent) were discharged to a nursing or rehabilitation facility. The 30-day mortality was 10.3 per cent and median observed LOS was 20 days. The frequencies of all other outcomes are presented in *Table 2*.

**Table 2:** Observed outcomes and predicted risk

Outcome	Observed	*n* (%)	Predicted risk (%), median (range)	*P*	SBS (%)
Any complication	Positive	146 (36)	28.7 (15.2–54.3)	*<0.001*	4.97
	Negative	262 (64)	25.5 (14.7–51.3)		
Serious complication	Positive	130 (32)	26 (12.7–49)	*<0.001*	4.83
	Negative	278 (68)	22.8 (12–46.7)		
Discharge to nursing or rehab. facility	Positive	86 (21)	16.5 (1–58.2)	*<0.001*	8.46
	Negative	322 (79)	8.5 (0.9–56.7)		
Return to operating room	Positive	84 (21)	7.1 (4.8–11.8)	*0.027*	1.37
	Negative	324 (79)	6.7 (4.1–13)		
Delayed gastric emptying	Positive	77 (19)	15.4 (9.2–30.4)	0.243	<1
	Negative	331 (81)	15.2 (7.9–39.7)		
Surgical site infection	Positive	50 (12)	13.75 (9–20.1)	*0.017*	1.76
	Negative	358 (88)	12.35 (6.9–22.7)		
Death	Positive	42 (10)	2.25 (0.1–13.6)	*0.001*	3.77
	Negative	366 (90)	0.8 (0–24.2)		
Pneumonia	Positive	32 (8)	7.65 (0.8–16.1)	*<0.001*	4.62
	Negative	376 (92)	4.5 (0.6–24.5)		
Renal failure	Positive	28 (7)	1.9 (0–4.9)	*0.024*	1.99
	Negative	380 (93)	1.3 (0–7)		
Re-admission	Positive	25 (6)	16.7 (12 -26.5)	0.127	<1
	Negative	383 (94)	15.8 (8.4–37.1)		
Cardiac complication	Positive	5 (1)	5.6 (4.9–9.6)	*0.002*	<1
	Negative	403 (99)	2.5 (0.1–15.6)		
Venous thromboembolism	Positive	5 (1)	3.8 (1.9–5.2)	0.256	<1
	Negative	403 (99)	3.2 (1.4–6.4)		

*P*-values were calculated using Mann–Whitney *U* test. Scaled Brier score (SBS) of the logistic regression model with 100 per cent indicating a perfect prediction. *P* ≤ 0.050 are italic. Further details on SBS are described in the *Supplementary material*.

### Predicted *versus* observed outcomes

For all complications except re-admission (*P* = 0.127), delayed gastric emptying (*P* = 0.243), and venous thromboembolism (*P* = 0.256), the PR significantly differed between positive and negative patients. Highly significant differences were observed for mortality, discharge to rehabilitation or nursing facility, cardiac complications, and pneumonia (all *P* ≤ 0.002). Prediction of outcome categories also differed significantly between positive and negative patients (both *P* < 0.001). For all outcomes, the mean PR was higher in positive patients compared with patients without complications. The actual PR was below 50 per cent in almost all cases. The SBS was highest for discharge to rehabilitation (8.46 per cent). Prediction of death, any complication, serious complication, and pneumonia scored between 3.77 and 4.97 per cent. For all other outcomes SBS was calculated below 2 per cent. *Table 2* compares average PRs for positive and negative patients and shows SBS for each outcome.

### Chance of outcome

Categorization of patients into below average, average, and above average COO showed highly significant variation between positive and negative patients for discharge to rehab facility (*P* < 0.001), renal failure (*P* = 0.003), pneumonia (*P* = 0.001), serious complication, and any complication (both *P* < 0.001). Variation in COO for mortality showed a weak significance (*P* = 0.050). For all other outcomes, no significant difference in COO between positive and negative patients was observed. *Table 3* shows contingency tables for COO *versus* positive and negative patients for each outcome.

**Table 3:** Chance of outcome

Outcome	Observed	Chance of outcome	*n* (%)	*P*
Any complication	Positive	Above average	43 (29.5)	*<0.001*
		Average	58 (39.7)	
		Below average	45 (30.8)	
	Negative	Above average	39 (14.9)	
		Average	89 (34.0)	
		Below average	134 (51.1)	
Serious complication	Positive	Above average	32 (37.2)	*0.001*
		Average	29 (33.7)	
		Below average	25 (29.1)	
	Negative	Above average	65 (20.2)	
		Average	106 (32.9)	
		Below average	151 (46.9)	
Discharge to nursing or rehab. facility	Positive	Above average	67 (77.9)	*<0.001*
		Average	6 (7.0)	
		Below average	13 (15.1)	
	Negative	Above average	156 (48.4)	
		Average	32 (9.9)	
		Below average	134 (41.6)	
Return to operating room	Positive	Above average	22 (26.2)	0.135
		Average	32 (38.1)	
		Below average	30 (35.7)	
	Negative	Above average	57 (17.6)	
		Average	120 (37.0)	
		Below average	147 (45.4)	
Delayed gastric emptying	Positive	Above average	33 (42.9)	0.568
		Average	22 (28.6)	
		Below average	22 (28.6)	
	Negative	Above average	121 (36.6)	
		Average	100 (30.2)	
		Below average	110 (33.2)	
Surgical site infection	Positive	Above average	18 (36.0)	0.287
		Average	18 (36.0)	
		Below average	14 (28.0)	
	Negative	Above average	92 (25.7)	
		Average	140 (39.1)	
		Below average	126 (35.2)	
Death	Positive	Above average	23 (54.8)	*0.050*
		Average	2 (4.8)	
		Below average	17 (40.5)	
	Negative	Above average	137 (59.0)	
		Average	13 (3.6)	
		Below average	216 (37.4)	
Pneumonia	Positive	Above average	24 (75.0)	*0.001*
		Average	3 (9.4)	
		Below average	5 (15.6)	
	Negative	Above average	153 (40.7)	
		Average	54 (14.4)	
		Below average	169 (44.9)	
Renal failure	Positive	Above average	20 (74.1)	*0.002*
		Average	0 (0)	
		Below average	7 (25.9)	
	Negative	Above average	156 (41.4)	
		Average	42 (11.1)	
		Below average	179 (47.5)	
Re-admission	Positive	Above average	3 (12.0)	0.099
		Average	13 (52.0)	
		Below average	9 (36.0)	
	Negative	Above average	57 (14.9)	
		Average	120 (31.3)	
		Below average	206 (53.8)	
Cardiac complication	Positive	Above average	5 (100.0)	0.08
		Average	0 (0.0)	
		Below average	0 (0.0)	
	Negative	Above average	190 (47.1)	
		Average	42 (10.4)	
		below average	171 (42.4)	
Venous thromboembolism	Positive	Above average	3 (60.0)	1
		Average	1 (20.0)	
		Below average	1 (20.0)	
	Negative	Above average	174 (43.2)	
		Average	114 (28.3)	
		Below average	115 (28.5)	

The 3 × 2 contingency tables are shown for each outcome, grouping patients for chance of outcome as provided by the American College of Surgeons surgical risk calculator (below, average and above average risk) and observed outcome (positive *versus* negative). Distributions were analysed using Pearson’s chi-squared test for expected values more than 5 and Fisher’s exact test for expected values less than 5. *P* ≤ 0.050 are italic.

### Length of stay

Median observed LOS was 19.5 days for patient discharged at home (208). Predicted LOS by the ACS SRC was significantly lower at 9.5 days (*P* < 0.001). Including all 408 patients regardless of discharge procedure, median observed LOS was 20 days and median predicted LOS was 10 days (*P* < 0.001).

Further analysis results are presented in *Table S3*.

## Discussion

This study aimed to evaluate performance of the ACS SRC in an external non-validated patient collective. Today a surgeon may consult a multitude of risk calculators, scores, or predictive algorithms before attempting a procedure. On one end of the spectrum are SRSs limited to certain procedures and outcomes (such as the pancreatic fistula risk score)^18^. On the other end are procedure-unspecific risk calculators that predict numerous outcomes at once (such as ACS SRC). Furthermore, tools may be divided into ones that rely solely on preoperative available information (such as ACS SRC, Combined Assessment of Risk Encountered in Surgery (CARES), ASA, Predictive OpTimal Trees in Emergency Surgery Risk (POTTER), and Score Predicting Early Mortality (SPEM)) and others which depend at least in part on intraoperatively available information (such as POSSUM, fistula risk score, and SAS)^4,5,18–20^.

Even though pre-dated by procedure- or disease-specific risk scores and calculators, the ACS SRC was the first available universal SRC^3^. Other more recently developed tools include the CARES SRC and the POTTER calculator^4,5^.

The ACS SRC assigned a higher median PR to positive patients for nine of 12 predicted outcomes (*Fig. 2*). The authors therefore expected the risk factors evaluated by the ACS SRC to correctly lead to higher PRs for these outcomes. PRs are expressed by the ACS SRC in three categories of COO as described above. However, cut-off values for sorting PRs into COO categories are not publicly available^21^. In contrast to median calculated PRs, it was found that only five outcomes (discharge, renal failure, pneumonia, serious complication, and any complication) showed significant differences of COO between positive and negative outcomes (*Table 3*). Summarizing these results, the ACS SRC correctly predicted a higher median PR for positive patients in 75 per cent of predicted outcomes; however, the higher median PR was correctly classified as an above average COO in only 42 per cent of outcome categories.

Outcome prediction by SRCs on a per patient percentage basis can be evaluated following varying approaches. Using a frequentist approach, one would expect a perfect SRC to assign 100 per cent risk to positive patients and 0 per cent risk to negative patients. This would lead to a SBS of 100 per cent^22^. As no outcome prediction reached a SBS above 8.5 per cent in our study, we found actual PR numbers by the ACS SRC to be a poor predictor of outcomes from the individual patient’s perspective (*Table 2*)^17^.

The ACS SRC being unable to make a perfect prediction, at least from a frequentist perspective, has already been acknowledged by its authors. Nonetheless, predicted risks by the SRC may be useful in a clinical setting. Even though experienced surgeons are capable of predicting some adverse outcomes after emergency surgery with similar discrimination as risk calculators, high estimated risks may raise awareness for specific outcomes or in patients presenting with a multitude of seemingly less-severe co-morbidities^23^. Additional information by the SRC could help the surgeon in adapting their approach to mitigate specific negative outcomes (for example, less aggressive resection, extended prehabilitation, or duration of postoperative surveillance). Considering this, interpretation of results not as a numeric risk but as a COO (as also provided by the SRC) might be more useful in clinical practice. Unfortunately, in this study, categorization of patients into different COO groups did not significantly correlate with positive outcomes for the majority of outcome categories (*Table 3*).

Multiple approach-related parameters are known to influence outcomes in surgery. These include, among others, indication of the procedure, one- or two-step resections, hospital volume, surgeon experience, minimally invasive approach, vascular or additional organ resection, estimated blood loss, and more^20,24–26^. Unfortunately, these factors cannot be specifically accounted for in the ACS SRC. In this study, the aim was to reduce the influence of surgical technique by evaluating a procedure without a pancreatic anastomosis. One option to adjust predicted outcomes for risk factors not included in the ACS SRC is the use of the SAR; however, SAR should be used with caution according to the SRC’s documentation as it increases all risks that are closer to a procedure-specific baseline risk than two s.d. On the other hand, predicted outcomes with a risk higher than two s.d. from baseline cannot be altered by SAR.

Since its release in 2013, multiple studies aimed to validate the ACS SRC using various collectives^14^. Studies using the NSQIP collective (from which the ACS SRC was derived) found good performance in predicting complications after pancreatic head and colorectal resections^10–12^. For collectives outside the NSQIP, studies showed mixed results.^7–9,13,18^ Statistical methods used for performance assessment varied between all above-mentioned studies. This was most apparent for the interpretation of COO as studies grouped COO classes differently to predict outcomes.

Reacting to these findings, the ACS SRC’s authors argue that only heterogenous multicentric collectives allow for a valid examination of performance. The ACS SRC only adjusts a baseline for patient-specific risk factors and leaves out confounders such as different surgical techniques or case volume per hospital. The authors of the current study tried to keep these to a minimum by evaluating a high-risk procedure with less room for variation compared with, for example, pancreatic resection with anastomosis. Using large samples from the NSQIP database they demonstrated that samples including more than 100 positive patients are necessary for performance assessment^14^. Given an expected occurrence rate of 4 per cent for an outcome this would require a data set of at least 2500 patients. As total pancreatectomy has a high rate of morbidity, it was hoped to be able to produce valid results using a smaller sample^27^. As requests for publication of the detailed model of the ACS SRC have been rejected, automated external validation remains impossible^21^. Given the large amounts of data necessary, one could argue whether an external validation, meeting the above-described criteria, may not be feasible at all.

The present study has several limitations that warrant emphasis. First, using data from the StuDoQ|Pancreas Registry, patients were already preselected for being operated on in a participating hospital. Therefore, this study only allows for assessment of the ACS SRC regarding this collective. Another limitation is that only the outcomes of morbidity and serious complication included more than 100 positive patients. As shown by Cohen *et al.*, validation of the ACS SRC benefits from data sets with more than 100 positive cases for a given outcome^14^. Especially for outcomes with fewer than 50 positive patients, the validity of these findings is questionable according to the ACS SRC’s developers.^14^ Finally, not all data from the StuDoQ|Registry exactly matched the ACS SRC’s definition. In some cases, risk factors or observed outcomes had to be synthesized from other variables. Entering preoperative sepsis and ventilation as not present for all patients could have biased the PR towards lower percentages.

In conclusion, while SRCs can provide meaningful information for surgeons and patients, the ACS SRC did not adequately predict outcomes after total pancreatectomy in this collective. Surgeons using the ACS SRC for predictions in non-validated collectives should be aware that its accuracy might be drastically reduced. Without access to its model, external validation of the ACS SRC remains technically challenging. Whether a newly developed SRC based on StuDoQ registry data would lead to better performance remains unclear.

## Supplementary Material

zrac164_Supplementary_Data

## Data Availability

Original data can be provided on request and after a positive vote by the StuDoQ steering committee. An exemplary R script for evaluation of prediction of postoperative mortality can be provided on request.
