# Supplementary material for: Applicability of the surgical risk calculator by the American College of Surgeons in the setting of German patients undergoing complete pancreatectomy: multicentre study using data from the StuDoQ|Pancreas registry
Source: BJS Open. 2023 Mar 7;7(2):zrac164. doi: 10.1093/bjsopen/zrac164 (PMC9991503; doi:10.1093/bjsopen/zrac164)
Supplement: zrac164_Supplementary_Data [file zrac164_supplementary_data.docx]

Applicability of the Surgical Risk Calculator by the American College of Surgeons in the setting of German patients undergoing complete pancreatectomy: A multicenter study using data from the StuDoQ|Pancreas registry

Philipp Höhn¹, Fabian Runde^2^, Andreas Minh Luu¹, Tim Fahlbusch¹, Daniel Fein¹, Carsten Klinger^3^, Waldemar Uhl¹, Orlin Belyaev¹ and members of StuDoQ|Pancreas registry of the German Society for General and Visceral Surgery (DGAV)*

* Tobias Keck^4^, Jens Werner^5^, Natascha Nüssler^6^, Detlef K. Bartsch^7^, Christoph-Thomas Germer^8^, Helmut Friess^9^, Christian Mönch^10^, Karl-Jürgen Oldhafer^11^, Jörg C. Kalff^12^

¹Department of General and Visceral Surgery, St. Josef-Hospital, Ruhr-Universität Bochum, Bochum, Germany

^2^Faculty of Medicine, Ruhr-Universität Bochum, Bochum, Germany

^3^German Society for General and Visceral Surgery, Berlin

^4^Surgical Department, Universitätsklinikum Schleswig-Holstein Campus Lübeck, Germany

^5^Department of General, Visceral, Transplant, Vascular and Thoracic Surgery, Klinikum Großhadern, Ludwig-Maximilians-Universität München, Germany

^6^Department of General and Visceral Surgery, Endocrine Surgery und Coloproctology, Klinikum Neuperlach, Städt. Klinikum München GmbH, Germany

^7^Department of Visceral, Thoracic and Vascular Surgery, Universitätsklinikum Marburg, Germany

^8^Department of General, Visceral, Vascular and Pediatric Surgery, Universitätsklinik Würzburg, Germany

^9^Department of Surgery, Klinikum rechts der Isar, Technische Universität München, Germany

^10^Department of General, Visceral and Transplant Surgery, Westpfalz-Klinikum Kaiserslautern, Germany

^11^Department of Surgery, Asklepios Klinik Barmbek, Germany

^12^Department of Visceral, Colorectal Surgery and Proctology, Universitätsklinikum Bonn, Germany

**Corresponding Author:**

Dr. med. Philipp Höhn

Department of General and Visceral Surgery

St. Josef-Hospital

Gudrunstr. 56

44791 Bochum

Philipp.hoehn@rub.de

+49 234 509 2211

Orcid-ID: 0000-0003-2913-9690

**Supplementary Materials - Index**

| Supplementary Methods |  |
| --- | --- |
| Data synthesis | pag. 3 |
| Data analysis | pag. 4 |
| Supplementary Results |  |
| Test statistics and effect sizes | pag. 5 |
| Supplementary Appendixes |  |
| Appendix S1: Department heads of surgical clinics which provided study data | pag. 6 |
| Supplementary Figures and Tables |  |
| Table S1: Preoperative risk factor definitions | pag. 8 |
| Table S2: Differing outcome definitions  Table S3: Brier scores and effect sizes | pag. 10  pag. 11 |
|  |  |

**Supplementary Methods**

*Data synthesis*

The ACS SRC accepts categorical data for 18 of 20 preoperative risk factors. Of these 18, 12 are binary variables. Height and weight are accepted as numeric variables. Detailed definitions on each category are provided by the frontend. We compared the definition for each variable with definitions by the StuDoQ|Pancreas registry if the same risk factor was recorded. Definition of age, sex group, functional status, emergency case, Steroid use for chronic condition, ascites within 30 days prior to surgery, disseminated cancer, diabetes, hypertension requiring medication, ASA class, history of severe COPD and dialysis was identical between ACS SRC and StuDoQ|Pancreas. Therefore, these risk factors as well as height and weight were entered into the ACS SRC as provided by the registry. Data on congestive heart failure in 30 days prior to surgery, dyspnea, current smoker within 1 year and acute renal failure was provided by StuDoQ|Pancreas but definitions differed from ACS SRC categories. Values for these risk factors were derived by other data provided by the registry (e. g. data for preoperative dyspnea was derived from NYHA classes documented by StuDoQ|Pancreas). Information on preoperative systemic sepsis and ventilator dependency could not be derived from the registry. We assumed that patients with these conditions would be deemed unfit for elective total pancreatectomy. As the ACS SRC requires a yes or no entry, these risk factors were categorically entered as not present.

Postoperative outcomes were similarly processed. Appearance of SSI, Death, Discharge to nursing or rehabilitation facility as well as delayed gastric emptying (DGE) was recorded by the registry according to the ACS SRC’s definitions allowing for direct comparison. Data on postoperative sepsis and UTI was not provided by StuDoQ|Pancreas and outcomes therefore excluded from analysis. Data on all other outcomes was provided by the registry but definitions differed between StuDoQ|Pancreas and ACS SRC. In these cases, binary outcome data was derived from other information provided by StuDoQ|Pancreas.

Detailed definitions, as well as data synthesis algorithms, are provided in supplementary table 1 for preoperative risk factors and supplementary table 2 for postoperative outcomes.

*Data analysis*

Validity of PR was evaluated using a binary logistic regression model, PR being the dependent and observed outcome being the independent binary categorical variable. Similar to previous publications, we used a Brier-Score to evaluate our model’s accuracy. The Brier Score is defined as the mean squared difference between PR and either 0 (negative outcome) or 1 (positive outcome). This leads to a Brier Score approaching zero for perfect accuracy. Maximum values (Brier_max_) are dependent on the observed outcome’s incidence (e. g. 0.25 for an incidence of 50 %), indicating a random prediction. We used a Brier Score scaled to its incidence dependent maximum value to account for the low incidence observed for multiple outcomes (SBS). SBS is calculated using the non-scaled Brier Score and the average PR of each outcome as shown below. A SBS of 100 % indicates a perfection prediction.

$SBS=1-\frac{Brier}{{Brier}_{max}}$ ${Brier}_{max}= \bar{p}*(1- \bar{p}$)

$$p=predicted risk for each patient$$

**Supplementary Results**

Test statistics and effect sizes of Mann-Whitney U and χ²-tests are shown in supplementary table 3. All significant differences between central tendencies showed weak to moderate effects (pearson correlation coefficient 0.11 – 0.3). Non scaled Brier Scores are also presented in supplementary table 3. An exemplary R script for evaluation of prediction of postoperative mortality can be provided on request.

**Supplementary Appendixes**

*Appendix S1: Department heads of surgical clinics which provided study data*

Carsten Gutt (Allgemein-, Visceral-, Gefäß- und Thoraxchirurgie, Klinikum Memmingen), Jörg Köninger (Allgemein- und Visceralchirurgie, Katharinenhospital), Andreas Schnitzbauer (Kllinik für Allgemein- und Viszeralchirurgie, Universitätsklinikum Frankfurt), Clemens Schafmayer (Allgemeine-, Thorax-, Gefäß- und Transplantationschirurgie, Universitätsmedizin Rostock), Stefan Farkas (Klinik für Allgemein- und Visceralchirurgie, St.-Josefs-Hospital Wiesbaden), Werner Hartwig (Klinik für Allgemein-, Viszeral- und Gefäßchirurgie, EvK Düsseldorf), Sören Torge Mees (Allgemein- und Viszeralchirurgie, Städtisches Klinikum Dresden Friedrichstadt), Frank Klammer (Allgemein-, Viszeral- und Thoraxchirurgie, St. Franziskus-Hospital), Matthias Glanemann (Klinik für Allgemein-, Viszeral-, Gefäß- und Kinderchirurgie, Universitätsklinikum des Saarlandes), Michael Ghadimi (Klinik für Allgemein-, Viszeral- und Kinderchirurgie, Universitätsmedizin Göttingen), Matthias Anthuber (Klinik für Allgemein-, Viszeral- und Transplantationschirurgie, Klinikum Augsburg), Christoph Reißfelder (Chirurgische Klinik, Universitätsmedizin Mannheim), Pompiliu Piso (Chirurgie I, Barmherzige Brüder Regensburg), Winfried Padberg (Allgemein-, Viszeral-, Thorax-, Tranplantations- und Kinderchirurgie, Universitätsklinikum Gießen Marburg Standort Gießen), Robert Grützmann (Chirurgische Klinik, Universitätsklinikum Erlangen), Marco Niedergethmann (Klinik für Allgemein- und Viszeralchirurgie, Alfried-Krupp-Krankenhaus), Andreas Pascher (Klinik und Poliklinik für Allgemein- und Viszeralchirurgie, Universitätsklinikum Münster), Klaus Prenzel (Allgemein- und Viszeralchirurgie, Marienhausklinik Ahrweiler), Hans-Bernd Reith (Allgemein- und Viszeralchirurgie, Agaplesion Diakoniekliniken Kassel), Ansgar Michael Chromik (Abteilung für Allgemein- und Viszeralchirurgie, Asklepios Klinikum Harburg), Colin M. Krüger (Chirurgie, Immanuelklinik Rüdersdorf), Hüseyin Bektas (Klinik für Allgemein-, Viszeral- und onkologische Chirurgie, Klinikum Bremen-Mitte), Bertram Illert (Klinik für Allgemein- und Viszeralchirurgie, Sana Kliniken Lübeck GmbH), Merten Hommann (Allgemeine Chirurgie, Viszeralchirurgie, Zentralklinik Bad Berka), Jörg-Peter Ritz (Klinik für Allgemein- und Viszeralchirurgie, Helios Kliniken Schwerin), Axel Döhrmann (Klinik für Allgemein- und Viszeralchirurgie, Katholisches Klinikum Oberhausen), Nico Schäfer (Klinik für Allgemein-, Visceral- und Thoraxchirurgie, Klinikum Leverkusen), Thomas Kraus (Allgemeine, Viszeral und Minimalinvasive Chirurgie, Krankenhaus Nordwest GmbH), Mark Jäger (Klinik für Allgemein-, Viszeral- und MIC, Städtisches Klinikum Wolfenbüttel gGmbH), Jörg Tschmelitsch (Chirurgie, Barmherzige Brüder St. Veit), Ullrich Fleck (Allgemeinchirurgie/ Viszeralchirurgie, DRK-Krankenhaus Luckenwalde), Michael Pauthner (Chirurgische Klinik I, Sana Klinikum Offenbach), Ute Tröbs (Allgemein- und Viszeralchirurgie, Kreiskrankenhaus Delitzsch), Albrecht Stier (Klinik für Allgemein- und Viszeralchirurgie, Helios-Klinik Erfurt), Carsten Krones (Klinik für Allgemein- und Viszeralchirurgie, Marienhospital Aachen)

**Supplementary Figures and Tables**

*Table S1: Preoperative risk factor definitions*

| Risk factor | StuDoQ | ACS SRC |
| --- | --- | --- |
| **Functional Status** | - Independent: Karnofsky-Index 100%-70% - Partially dependent: Karnofsky-Index 60%-40% - Totally dependent: Karnofsky-Index 30%-10% | The best functional status/level of self-care demonstrated by the patient within the 30 days prior surgery.   - Independent: The patient does not require assistance from another person for any activities of daily living. This includes a person who is able to function independently with prosthetics, equipment, or devices. - Partially dependent: The patient requires some assistance from another person for activities of daily living. - Totally dependent: The patient requires total assistance for all activities of daily living. |
| **Emergency Case** | Case is reported as emergent by any available source. | The principal operative procedure must be performed during the hospital admission for the diagnosis AND the surgeon and/or anesthesiologist must report the case as emergent. |
| **Steroid use for chronic condition** | Oral or parenteral administration of more than 7.5 mg prednisolone equivalent per day for > 6 months discontinued less than 3 months before op. | Regular administration of oral or parenteral corticosteroid medications or immunosuppressants for a chronic medical condition, within the 30 days prior to surgery, or at the time the patient is being considered as a candidate for surgery. A one-time pulse, limited short course, or a taper of less than 10 days duration would not qualify. Long-interval injections of long-acting agents would qualify. |
| **Ascites within 30 days prior to surgery** | Documented ascites within 30 days prior to surgery by any source. | The presence of fluid accumulation in the peritoneal cavity noted on physical examination, abdominal ultrasound, or abdominal CT/MRI within 30 days prior to surgery. Documentation must state either active or a history of liver disease or must state secondary to malignancy. |
| **Disseminated Cancer** | Patients with metastases in multiple organs. | The patient has a primary cancer that has metastasized to a major organ AND meets at least one of the following:   - active treatment for the cancer within one year of the surgery date. If the surgical procedure is the treatment for the metastatic cancer, answer "Yes". - the patient has elected not to receive treatment for the metastatic disease - the patient's metastatic cancer has been deemed untreatable   Report the following cancers as Disseminated Cancer: Acute Lymphocytic Leukemia (ALL), Acute Myelogenous Leukemia (AML), and Stage IV Lymphoma. Do not report the following as Disseminated Cancer: Chronic Lymphocytic Leukemia (CLL), Chronic Myelogenous Leukemia (CML), Stages I through III Lymphomas or Multiple Myeloma. |
| **Diabetes** | All insulin dependent and non-insulin dependent diabetes. | - Oral - Insulin   The individual requires daily dosages of exogenous parenteral insulin or an oral hypoglycemic agent to prevent a hyperglycemia. A patient is not included if diabetes is controlled by diet alone. |
| **Hypertension requiring medication** | Any form of antihypertensive medication. | The patient has a diagnosis of HTN in the medical record and will require antihypertensive medication(s) within 30 days prior to surgery. |
| **History of Severe COPD** | Any form of chronic bronchodilator therapy or any symptomatic COPD (e. g. dyspnea). Does not include patients whose only pulmonary disease is asthma | Chronic obstructive pulmonary disease (such as emphysema and/or chronic bronchitis) resulting in one or more of the following:   - Functional disability from COPD (for example, dyspnea, inability to perform ADLs) - Hospitalization in the past for treatment of COPD - Chronic bronchodilator therapy with oral or inhaled agents - FEV1 of <75% of predicted - Do not include patients whose only pulmonary disease is asthma   Do not include patients with diffuse interstitial fibrosis or sarcoidosis |
| **Dialysis** | Preoperative renal failure requiring regular dialysis (e. g. hemodialysis, peritoneal dialysis) | Acute or chronic renal failure requiring treatment with peritoneal dialysis, hemodialysis, hemofiltration, hemodiafiltration, or ultrafiltration within 2 weeks prior to surgery. If a patient requires dialysis, but refuses it, the answer to this variable will be "Yes." |
| **Congestive Heart Failure in 30 days prior to surgery** | NYHA class is recorded.  For Patients NYHA > II, CHF was entered as positive. | Only newly diagnosed CHF within the previous 30 days or a diagnosis of chronic CHF with signs or symptoms of CHF in the 30 days prior to surgery fulfills this definition. |
| **Dyspnea** | Not recorded  NYHA = III was entered as dyspnea with moderate exertion.  NYHA = IV was entered as dyspnea at rest. | The patient's dyspnea status when they were in their usual state of health, prior to the onset of the acute illness, within the 30 days prior to the time the patient is being considered a candidate for surgery.   - No - With moderate exertion - At rest |
| **Current Smoker within 1 Year** | Packet years are recorded. If the patient ceased smoking, years between last cigarette and surgery are recorded.  Patient were entered as smoking if:   - They were recorded as current smoker or stopped smoking < 1 year - > 20 PY were recorded and current status or last cigarette were not recorded | The patient has smoked cigarettes in the year prior to admission for surgery. Patients who smoke cigars or pipes or use chewing tobacco are not included. |
| **Acute Renal Failure** | Preoperative acute renal failure is not recorded as an independent variable. Patients were entered as positive if recorded serum creatinin > 3 mg/dl was recorded within 30 days prior to surgery. | A clinical condition associate with rapid decline of kidney function. The patient meets one of the following:   - Increased BUN on two measurements AND two Cr results > 3mg/dl - Surgeon or physician has documented Acute Renal Failure AND one of the following:   - Increased BUN on two measurements   - Two Cr results > 3mg/dl |
| **Systemic Sepsis within 48 hours prior to surgery** | Not available. All patients were entered as negative. | Any of the following occurring within 48 hours prior to surgery:   - Systemic Inflammatory Response Syndrome (SIRS) - Sepsis - Septic Shock |
| **Ventilator Dependent** | Not available. All patients were entered as negative. | A patient requiring ventilator-assisted respiration at any time during the 48 hours preceding surgery. This does not include the treatment of sleep apnea with CPAP. |

Diverting definitions of preoperative risk factors are compared between StuDoQ|Pancreas Registry and ACS SRC. Bullet point lists mark categorical variables accepted by ACS SRC. All other variables only allow for binary entry.

*Table S2: Differing outcome definitions*

| Outcome | StuDoQ | ACS SRC |
| --- | --- | --- |
| **Any Complication** | Outcome was observed if any of the following complications were recorded:  Pneumonia, myocardial infarction, pulmonary embolism, any type of surgical site infection, wound disruption, unplanned ventilation > 24 hours, stroke, acute rental failure, return to the operating room or DVT. | Additionally includes the outcomes cardiac arrest, systemic sepsis, unplanned intubation and UTI which are not recorded by StuDoQ\|Pancreas |
| **Serious Complication** | Outcome was observed if any of the following complications were recorded:  Pneumonia, myocardial infarction, pulmonary embolism, deep incisional SSI, organ space SSI, wound disruption, acute rental failure, return to the operating room or DVT | Additionally includes the outcomes cardiac arrest, systemic sepsis, unplanned intubation and UTI which are not recorded by StuDoQ\|Pancreas |
| **Return to Operating Room** | Unplanned re-operation in narcosis. | Return to the operating room for additional surgery that was not planned at the time of the initial surgery. |
| **Pneumonia** | Infection of the lungs diagnosed by radiologic or clinical criteria. | Infection of the lungs, diagnosed using both radiologic (i.e., infiltrate, consolidation or opacity, cavitation) and clinical (e.g., fever, leukopenia/leukocytosis, culture results, patient symptoms) criteria. |
| **Renal failure** | Acute renal failure requiring dialysis | Includes either progressive renal insufficiency OR acute renal failure requiring dialysis   - Progressive renal insufficiency: a rise in creatinine of >2 mg/dl from preoperative value, but with no requirement for dialysis. - Acute renal failure requiring dialysis: A patient who did not require dialysis preoperatively, worsening of renal dysfunction postoperatively requiring hemodialysis, peritoneal dialysis, hemofiltration, hemodiafiltration, or ultrafiltration. |
| **Cardiac complication** | Postoperative myocardial infarction.  Includes transmural infarction but not temporary troponin elevation. | Includes cardiac arrest or myocardial infarction.   - Cardiac arrest: The absence of cardiac rhythm or presence of a chaotic cardiac rhythm requiring the initiation of CPR, which includes chest compressions. - Myocardial infarction: ECG changes, new elevation in troponin, or physician diagnosis. |
| **Venous thromboembolism** | Radiologically or clinically confirmed pulmonary embolism. | The identification of a new thrombus within the venous system, described in studies as present in the superficial or deep venous systems but requires therapy.  This diagnosis is confirmed by a duplex, venogram, CT scan or other imaging modality, AND the patient requires treatment with anticoagulation therapy and/or placement of a vena cava filter or clipping of the vena cava. |

Diverting definitions of outcomes are compared between StuDoQ|Pancreas Registry and ACS SRC. Any complication and serious complication were recorded as observed if any outcome, as defined by StuDoQ|Pancreas definition, was observed.

*Table S3: Brier scores and effect sizes*

| **Outcome** | **Effect size** | **Brier-Score** |
| --- | --- | --- |
| Any Complication | 0,218 | 0,218 |
| Serious Complication | 0,217 | 0,207 |
| Discharge | 0,297 | 0,152 |
| Return to OR | 0,11 | 0,161 |
| Delayed Gastric Emptying | 0,058 | 0,152 |
| Surgical Site Infection | 0,119 | 0,106 |
| Death | 0,171 | 0,09 |
| Pneumonia | 0,219 | 0,069 |
| Renal Failure | 0,112 | 0,063 |
| Readmission | 0,075 | 0,058 |
| Cardiac Complication | 0,156 | 0,012 |
| Venous Thromboembolism | 0,056 | 0,012 |

Effect sizes of Mann-Whitney U test are measured using Pearson’s correlation coefficient. 1 indicates a strong effect and 0 indicates no effect. Unscaled Brier scores show the mean squared difference between PR and either 0 (negative outcome) or 1 (positive outcome). Expecting an incidence of 50 %, a score of 0 indicates a perfect prediction and 0.25 a random prediction.
